# Practical needs and considerations for refugees and other forcibly displaced persons with neurological disorders: Recommendations using a modified Delphi approach

**DOI:** 10.12688/gatesopenres.13447.1

**Published:** 2021-12-14

**Authors:** Shawheen Rezaei, Foksouna Sakadi, Fu-Liong Hiew, Ildefonso Rodriguez-Leyva, Jera Kruja, Mohammad Wasay, Osheik AbuAsha Seidi, Saad Abdel-Aziz, Shahriar Nafissi, Farrah Mateen, Global Working Group for Refugees with Neurological Needs

**Affiliations:** 1 Neurology, Massachusetts General Hospital, Boston, Massachusetts, 02114, USA; 2 General Hospital of National Reference, N'Djamena, Chad; 3 Neurology, Kuala Lumpur Hospital, Kuala Lumpur, Malaysia; 4 Universidad Autonoma de San Luis Potosi, San Luis Potosi, Mexico; 5 Neurology, Hospital Central Dr. Ignacio Morones Prieto, San Luis Potosi, Mexico; 6 Neurology, University of Medicine, Tirana, Tirana, Albania; 7 Neurology, University Hospital Center Mother Teresa, Tirana, Albania; 8 Neurology, Aga Khan University Hospital, Karachi, Pakistan; 9 University of Khartoum, Khartoum, Sudan; 10 Neurology, Soba University Hospital, Khartoum, Sudan; 11 Médecins Sans Frontières, Amman, Jordan; 12 Neurology, Tehran University of Medical Sciences, Tehran, Iran; 13 Neurology, Shariati Hospital, Tehran, Iran; 14 Harvard Medical School, Boston, Massachusetts, 02115, USA

**Keywords:** Neurology; Refugee; Asylum; Armed Conflict; Epilepsy: Diagnosis; Practice; Stroke; Headache

## Abstract

*Background: *There are >70 million forcibly displaced people worldwide, including refugees, internally displaced persons, and asylum seekers. While the health needs of forcibly displaced people have been characterized in the literature, more still needs to be done globally to translate this knowledge into effective policies and actions, particularly in neurology.

*Methods:* In 2020, a global network of published experts on neurological disease and refugees was convened. Nine physician experts from nine countries (2 low, 1 lower-middle income, 5 upper-middle, 1 high income) with experience treating displaced people originating from 18 countries participated in three survey and two discussion rounds in accordance with the Delphi method.

*Results: *A consensus list of priority interventions for treating neurological conditions in displaced people was created, agnostic to cost considerations, with the ten highest ranking tests or treatments ranked as: computerized tomography scans, magnetic resonance imaging scans, levetiracetam, acetylsalicylic acid, carbamazepine, paracetamol, sodium valproate, basic blood tests, steroids and anti-tuberculous medication. The most important contextual considerations (100% consensus) were all economic and political, including the economic status of the displaced person’s country of origin, the host country, and the stage in the asylum seeking process. The annual cost to purchase the ten priority neurological interventions for the entire displaced population was estimated to be 220 million USD for medications and 4.2 billion USD for imaging and tests.

*Conclusions: *A need for neuroimaging and anti-seizure medications for forcibly displaced people was emphasized. These recommendations could guide future research and investment in neurological care for forcibly displaced people.

## Background

There are more than 70 million forcibly displaced people worldwide according to the United Nations High Commissioner for Refugees (UNHCR), a number that is unprecedented in the history of the organization ^1^. This figure includes 41.3 million internally displaced people (individuals fleeing their homes due to persecution, war or violence, but who still remain within their country’s border), 25.9 million refugees (individuals fleeing their country of origin due to persecution, war or violence, who have been granted refugee status under international law) and 3.5 million asylum seekers (individuals fleeing their country of origin due to persecution, war or violence, whose request for sanctuary has yet to be processed) ^1, 2^. Displaced people, due to the experiences of violence, famine, armed conflict and/or persecution associated with displacement, often face a set of challenges that are distinct from those of other patient populations ^3^. While the unique health needs of forcibly displaced people have been characterized in the literature, more still needs to be done on an international level to translate this knowledge into effective policies and actions, particularly in neurology ^4^.

Although people are fleeing their homes in the largest numbers since World War II, there is a documented lack of international collaboration to address the health needs of forcibly displaced populations ^3^. The neurologist community is poised to address this need through collaborative approaches. Especially in neurology, the treatment needs of forcibly displaced people are not well synthesized across locations and disorders. The published accounts available highlight the need for improved frameworks for understanding and treating the neurological conditions that come with displacement in more modern settings ^5– 7^. The determinants of neurological health are also being better recognized, noting that environmental conditions can further compound the lived experiences of refugees with neurological disorders. These other factors include - but are not limited to - malnutrition, climatic extremes, exposures to infections and toxins, lack of protections and security, poor sanitation, and generally unstable living conditions ^6– 9^. The neurological needs of displaced people often overlap mental health conditions resulting from the trauma and stress of displacement, including post-traumatic stress, anxiety and depressive disorders, calling for more in-depth investigations of the needs of this population in light of their specific context ^10– 12^. Furthermore, systemic hurdles ranging from the lack of contextualized medical education for refugees with neurological needs to the limited resources available in many countries of first asylum make quality healthcare less accessible to displaced people ^13– 15^.

By directly confronting this situation, the neurologists and related providers of neurological care have an opportunity to address the neurological toll of complex humanitarian emergencies ^16^. The development of a more complete vision for pragmatic actionable steps for neurological interventions will streamline healthcare systems and international actors approach to alleviating this high burden of disease. Although current care is reactive and responsive to needs as they arise, at best, future planning could lead to preparedness and avoidance of humanitarian crises compounding the burden of global neurological disease. 

Our modified Delphi consensus method attempts to address the critical need to devise a baseline set of guidelines on implementing concrete neurological interventions for displaced people in complex humanitarian emergencies ^17^.

## Methods

### The Delphi method

The Delphi method has been demonstrated to serve as an effective process for arriving at a consensus among a panel of experts in multiple disciplines ^18– 20^. Through an iterative process, experts are requested to complete surveys individually and then participate in group discussions to provide feedback on the results of the survey, which has been shown to enable a group to reach a consensus swiftly ^18^.

**Figure 1.**
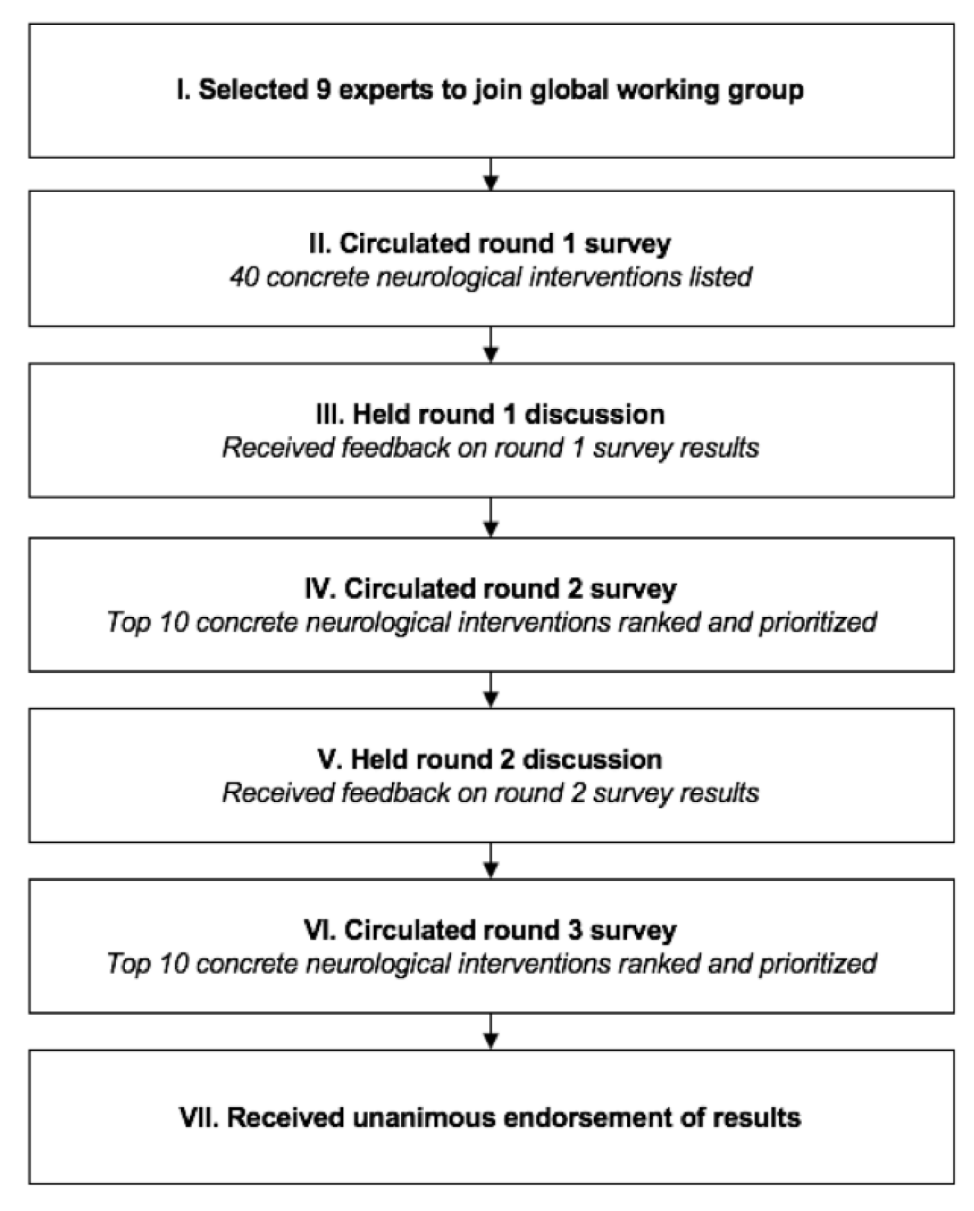
Delphi method flowchart.

An outline of the rounds of the modified Delphi method executed for this study is provided in Figure 1.

### Study objective and protocols

The project included two full rounds of surveying and discussion in accordance with the Delphi method, with a final round survey that determined the working group-endorsed lists of contextual considerations ( Table 1) and concrete neurological needs of displaced people globally ( Table 2). The study was deemed exempt from formal review by the Mass General Brigham Institutional Review Board.

**Table 1.** Ranking of other important considerations when treating neurological needs of displaced people.

Ranking	Consideration	Consensus (%)
1	Economic status of displaced person’s host country	100 *
2	Stage of displaced person in the asylum process	100 *
3	Economic status of displaced person’s country of origin	100 *
4	Availability of neurological care	63
5	Social support for displaced people	63
6	Language translation challenges	50
7	Rehabilitation facilities	50
8	Care of women and children	38
9	Healthcare coverage in national system for displaced persons	38
10	Education on how to care for displaced people	25
11	Increased training of neurologists	25
12	Mobile care teams/telemedicine	25
13	Placement upon discharge from hospital	25
14	Support of scientific study projects on neurological pathologies	25
15	Cultural differences	13
16	Logistics between hospital and asylum center	13
17	Professional support for physicians on the field	13
18	Religious issues	13
19	Ambulances	0
20	Food quality	0
21	Sports and recreational facilities	0
22	Trauma and injury prevention	0

*Consensus reached through round 1 discussion

**Table 2.** Complete ranked list of neurological interventions for displaced people.

Ranking	Intervention	Rating ^1^
1	CT scan	90.9
2	MRI scan	89.8
3	Levetiracetam	89.1
4	Acetylsalicylic acid	86.6
5	Carbamazepine	86.3
6	Paracetamol	81.8
7	Sodium valproate	81.7
8	Basic blood tests	80.7
9	Steroids	79.4
10	Combined anti-tuberculous medications	78.0
11	Clopidogrel	76.8
12	Lamotrigine	76.7
13	Electroencephalogram	76.6
14	Blood pressure screening equipment	74.4
15	Escitalopram	73.4
16	Propranolol	73.3
17	Amitriptyline	73.2
18	Folate	73.0
19	Vitamin B12	72.4
20	Vitamin B complex	72.1
21	NSAID	71.8
22	Phenytoin	71.1
23	Statin	70.9
24	Fluoxetine	70.6
25	Vitamin B1	68.8
26	Vitamin B6	68.6
27	Pregabalin	68.0
28	Gabapentin	67.9
29	Electromyogram-Nerve conduction study	67.8
30	Parenteral Acyclovir	66.7
31	Levodopa-Carbidopa	65.1
32	Lumbar puncture sets	63.9
33	Vitamin D	63.2
34	Warfarin	62.4
35	Intavenous Immunoglobulins	60.1
36	Hydrochlorothiazide	59.0
37	Opiate	52.6
38	Amantadine	48.4
39	Magnesium	45.3
40	Methylmalonic acid	37.3
41	Homocysteine	34.6

### Member selection

In early February 2020, a list of 30 experts, primarily neurologists but also including non-neurologist physicians, was formed through an internet search through medical journal databases and professional websites. Expertise was determined by the individual’s knowledge of the unique neurological needs of forcibly displaced people as demonstrated by research publications and self-reported clinical exposure to displaced populations and was not limited to neurologists. The population considered forcibly displaced for this study included people displaced due to persecution or conflict and excluded people displaced due to economic or climate reasons. This list was further narrowed to 16 experts based on their geographic location, which were invited to join the study. A Global Working Group of nine experts representing the Republic of Albania, the Republic of Chad, the Islamic Republic of Iran, the Hashemite Kingdom of Jordan, Malaysia, Mexico, the Islamic Republic of Pakistan, the Republic of the Sudan and the United States of America was formed ( Table 3). Reasons for nonparticipation of members of the initial 30 experts included insufficient exposure to displaced populations and an inability to participate regularly in the Delphi method process. The process did not specifically address the effects of Covid-19 but was carried out during the pandemic. Of the selected Global Working Group participants, six experts treat asylum seekers, nine treat refugees, and five treat internally displaced persons ( Table 3).

**Table 3.** Background information of Global Working Group participants ^2^.

Participant	Country of residence	Specialty	Affiliations	Treat asylum seekers	Treat refugees	Treat internally displaced persons	Primary countries of origin of displaced people treated
Farrah Mateen	USA	Associate Professor of Neurology	Massachusetts General Hospital; Harvard Medical School	yes	yes	yes	Guinea, Sierra Leone, Syria, Iraq, Mexico
Foksouna Sakadi	Chad	Neurologist	General Hospital of National Reference	yes	yes	no	Central African Republic, South Sudan, Sudan, Libya, Nigeria
Fu-Liong Hiew	Malaysia	Neurologist	Kuala Lumpur Hospital	yes	yes	no	Myanmar (Burma), Syria
Ildefonso Rodriguez-Leyva	Mexico	Professor of Neurology	Universidad Autonoma de San Luis Potosi; Hospital Central Dr. Ignacio Morones Prieto	yes	no	yes	Mexico, various countries in Central and South America
Jera Kruja	Albania	Professor of Neurology	University of Medicine, Tirana; University Hospital Center Mother Teresa	no	yes	no	Iran
Mohammad Wasay	Pakistan	Professor of Neurology	Aga Khan University Hospital	no	yes	yes	Afghanistan, Myanmar (Burma), Pakistan
Osheik AbuAsha Seidi	Sudan	Professor of Neurology	University of Khartoum, Soba University Hospital	yes	yes	yes	Eritrea, Ethiopia, Sudan, South Sudan
Saad Abdel-Aziz	Jordan	Physician; Public Health Specialist	Médecins Sans Frontières (MSF) Jordan; Johns Hopkins Bloomberg School of Public Health	yes	yes	no	Syria
Shahriar Nafissi	Iran	Professor of Neurology	Tehran University of Medical Sciences; Shariati Hospital	no	yes	yes	Afghanistan, Iraq, Iran

### Round one of the Delphi method

In mid-March, the round one survey was distributed to the Global Working Group. The survey requested each expert to provide their background information in addition to listing their experiences with displaced people. The main survey questions asked the experts to rank the top ten neurological interventions to address the neurological needs of displaced people, ignoring costs; to rank a list of demographic factors of displaced people that make the largest difference in providing neurological care; and to list other considerations that are important when assessing the needs of displaced people. The experts were informed that “concrete” refers to material interventions such as medical supplies (e.g.: phenobarbital, thiamine, head CT). Once all Global Working Group participants completed the round 1 survey, the results were circulated to the group. A total of 41 concrete neurological needs were listed and 20 important issues that were ancillary to the actual provision of a treatment were listed by the Global Working Group participants. In early April, the round one discussion was convened via a face-to-face Zoom call to review the results of the survey, provide rationale for interventions listed, and provide suggestions to incorporate into the next round of the Delphi method.

### Round two of the Delphi method

Based on the results from round one, 14 larger categories of neurological interventions were developed, under which 33 specific items were listed after feedback. The experts were then asked to select and prioritize a list of ten interventions from the total list of interventions established from the previous round. The experts were also asked to rank the interventions from 1–4 based on the degree of necessity (1- absolutely necessary to 4- nice to have). In late May, the round two discussion took place via a face-to-face Zoom call with seven of the experts to provide feedback on the results of the round two survey. A comprehensive list of specific items was established within each category, and the experts agreed to rate specific items instead of categories for the final survey.

### Round three of the Delphi method

In early June, the round three and final survey was circulated among the experts. It requested that each expert rate 41 specific items on a scale of 0–100 independently, with 0 being not necessary and 100 being absolutely necessary for the treatment of displaced people. The ratings for these 41 interventions were then averaged from across the working group and ranked from highest mean rating to lowest. For select categories (anti-seizure, anti-headache/pain, neuro-psychiatric and cardiovascular/neurovascular), each expert was asked to select the top priority medication when treating displaced people. The number 1 priority and number 2 priority items for each of these categories was documented based on the percent of working group members that reached consensus on a given intervention.

### Cost assessment

A cost assessment was conducted for the top ten interventions, using a basic economic cost estimation model. The estimation for these interventions is provided in Table 4. These cost estimations used the most affordable price points available in the pharmaceutical market through online searches of goodrx.com in June 2020. The assessed price points were individual pricings and do not take into account large-scale price negotiations that an international organization could leverage. An illustrative dosage for each medication was selected based on prescribing patterns that are common for treating neurological conditions in adults. This assessment projected the population of displaced people needing care based on estimations made by a variety of sources such as the Center for Disease Control (CDC) and the World Health Organization (WHO). For imaging interventions, the percentage of displaced people requiring care was projected using the average MRI and CT scan usage estimates of >20 European countries from Eurostat. These data were used since many other countries do not have data collected or readily available on these interventions.

**Table 4.** Highest ranked neurological interventions for displaced people.

Rank	Intervention	Rating ^3^	Estimated % of population in need	Annual cost estimation (1,000 $USD) ^4^
*Imaging and tests*				
1	CT scan	90.9	8	2,200,000
2	MRI scan	89.8	6	1,700,000
3	Basic blood tests ^5^	80.7	20	320,000
*Medications and supplements*				
1	Levetiracetam	89.1	0.4	31,000
2	Acetylsalicylic acid	86.6	0.24	3,100
3	Carbamazepine	86.3	0.4	47,000
4	Paracetamol	81.8	3	46,000
5	Sodium valproate	81.7	0.4	70,000
6	Steroids	79.4	0.001	38
7	Anti-tuberculous medication	78.0	0.13	24,000

## Results

### Ranking of supplemental considerations important for neurological care

Throughout the iterations of surveying and discussions, the Global Working Group determined important contextual considerations ancillary to the provision of neurological care for displaced people that were not concrete neurological interventions. During the round one discussion, consensus was reached that the economic status of the displaced person’s host country made the largest difference on the approach to providing neurological care to displaced people, followed by the stage of the displaced person in the asylum process and the economic status of the displaced person’s country of origin. A list of contextual considerations when providing neurological care to displaced people, ranked based on percent consensus that the intervention is a top consideration, is provided in Table 1.

### Top neurological interventions for displaced people

A comprehensive, ranked list of 41 concrete neurological interventions for displaced people determined through the Delphi method is provided in Table 2. The top ten concrete neurological interventions to treat displaced people are provided in Table 4. They include two imaging interventions (CT scans and MRI scans) and three anti-seizure medications (levetiracetam, carbamazepine and sodium valproate).

For select categories of neurological interventions, the Global Working Group was asked to select the one medication they would use to treat displaced people. Table 5 provides the results of this prioritization, which was determined for anti-seizure, anti-headache/pain, neuropsychiatric, and cardiovascular/neurovascular treatments, along with cost estimations.

**Table 5.** Top two priority treatments for select treatment categories.

Type of treatment	Top rated intervention	% consensus	Cost per pill ($USD)
Anti-seizure	1) Levetiracetam	44	0.15
	1) Carbamazepine	44	0.23
Anti-headache/pain	1) Ibuprofen ^6^	33	0.12
	1) Amitriptyline	33	0.17
	1) Paracetamol	33	0.03
Neuro-psychiatric	1) Escitalopram	67	0.24
	2) Fluoxetine	33	0.12
Cardiovascular/ neurovascular	1) Acetylsalicylic acid	75	0.05
	2) Clopidogrel	25	0.26

### Assessing the costs of the intervention

As calculated in June 2020, the annual cost of providing the most important neurological interventions for the entire displaced population would be 220 million USD for medications and 4.2 billion USD for imaging and tests. The cost estimations for each of the most important neurological interventions are provided in Table 4 along with an estimated percent of displaced people in need of each intervention. This paper assumes a monotherapy anti-seizure treatment. As such, an average of the annual cost estimations of the three anti-seizure medications in the top 10 list (levetiracetam, carbamazepine and sodium valproate) was used when calculating the total annual cost for medications.

## Discussion

Through this novel collaboration across several continents, the Global Working Group offers foundational recommendations on the most pressing neurological interventions. Though WHO guidelines for mostly psychiatric conditions have been released as the Mental Health Gap Action Programme (mhGAP), there are currently no registries, surveilance or standards for specifically treating the neurological needs of displaced people in humanitarian contexts ^21^. Calls for action such as the World Health Assembly’s Intersectional Global Action Plan on Epilepsy and Other Neurological Disorders indicate growing interest in addressing these needs ^22^.

We build upon previously conducted economic evaluations of the neurological treatment of displaced people, which have been limited to micro-scale cost assessments of specific populations ^23^. As our cost assessment of top concrete neurological interventions indicates, the complete coverage of major neurological treatments among displaced people (such as seizures and chronic pain) would be relatively inexpensive. Especially in light of the high degree of disability caused by neurological conditions, this cost assessment suggests that focusing resources toward treating such conditions could be cost-effective and also dramatically improve the overall wellbeing of many displaced people. It is also important to stress that the price points for these interventions may in fact be much lower in the context of treating displaced people, since much of this population resides in lower income countries where the costs of production are much lower than higher income countries, where data on medication and other procedures are more readily available. This price difference would particularly be important to consider in the case of MRI scans, CT scans, and basic blood tests, where the only data available factor in the cost of labor. This cost is much higher in a high-income country context and consequently may not accurately reflect the true cost that would be incurred in the context in which a displaced person is treated. Furthermore, this finding could also suggest that more cost-effective ways of implementing these procedures for displaced people-- such as the provision of portable MRIs currently being piloted in low-income contexts-- should be explored to ensure that a minimum viable procedure is in place for displaced persons, if the status quo is too expensive to execute ^24– 26^.

### Study design: strengths and limitations

There are several strengths to this study. There has not been an expert-proposed set of recommendations of top neurological interventions for displaced people to date. These results create a foundation for future research and engagement for neurological care for displaced people. The Delphi method enabled the Global Working Group members to respond not only from their personal expertise in treating the neurological needs of displaced people but also from the insights from other members, improving the collective decision-making process. The geographic breadth of the Global Working Group members’ countries of residence, coupled with the diverse nationalities and official statuses of the displaced people they treat, makes the scope of the results more broadly applicable across a wider array of contexts. By synthesizing the experiences of a representative sample of neurologists and other providers treating displaced people globally and providing an estimation for how much these interventions may cost, these results facilitate the action of international organizations and funders seeking to improve the condition of displaced people. The findings thus help fill a critical gap in the understanding of neurological needs of displaced people with a pragmatic and expert-sponsored set of concrete recommendations.

There are also several limitations to our approach. While the study incorporated the expertise of 8 neurologists and one public health expert who have direct experience treating and assessing the neurological conditions of displaced people, inclusion of experts from more countries and other healthcare professions (such as nursing or emergency relief staff) could have added a greater variety of perspectives. We chose to inquire the most appropriate interventions agnostic to cost; other approaches could have included cost as an important, pragmatic consideration. However, ignoring cost in selection of priorities allows the list to remain relevant as costs change for medications and technologies over time. Similarly, we could have instead asked about common symptoms rather than neurological diseases in general. Given that many medications and interventions are overlapping across diseases, the current approach was selected.

Similarly, the estimated costs for implementing top neurological interventions have many limitations, since there is a great degree of variation in the pricing of items based on the vendors within a certain country context and the availability of pricing data. Several of the interventions also have multiple dosages, which were not specified during the rounds of the Delphi method. This study also only considers the cost of a single MRI or CT scan and does not factor in the costs of establishing the infrastructure and maintenance required for such imaging.

## Future directions

There are several steps that could be taken in order to act upon these recommendations. Since several of the items selected as most important through the Delphi method process are preliminarily estimated to be relatively inexpensive, funders that support relief aid for displaced people could allocate a portion of their budget for neurological treatments according to these cost estimations. International organizations addressing the needs of displaced people must then source these items from vendors in the regions in which they operate and ensure that supply chains exist to meet the needs on the ground. Challenges with availability of certain medications in specific country contexts exist, in light of the differing Essential Medicine Lists across countries.

Beyond funding and acquiring the appropriate material interventions, organizations must also take into account the present shortage of neurology expertise in the frontlines of care provision. Aside from educating more healthcare providers on the diagnosis of basic neurological conditions or training more neurologists to specifically care for displaced people, relief organizations could also allocate a portion of their budgets to promote the access and use of telehealth technology to connect frontline aid workers providing direct care to displaced people with neurologists skilled in specialized diagnoses and determining the appropriate treatment plan ^27^. If these steps are taken to ensure the adequate sourcing of interventions for persons with neurological disorders in humanitarian settings, the international community could make great strides toward improving the situation for forcibly displaced people worldwide.

## Data availability

Figshare: Neurological Needs of Refugees Round 3 Survey. DOI: https://doi.org/10.6084/m9.figshare.17124125.v1.

Data are available under the terms of the Creative Commons Zero "No rights reserved" data waiver (CC BY 4.0 Public domain dedication).

## Footnotes

^1^ Rating on a 0–100 scale, with 0 being not necessary and 100 being absolutely necessary for treating displaced people

^2^ Ordered alphabetically by first name

^3^ Rating on a 0-100 scale, with 0 being not necessary and 100 being absolutely necessary for treating displaced people

^4^ Estimation of annual cost of standard dosage/procedure for the percent of displaced population in need of a given intervention

^5^ Complete blood panel used as example of basic blood test.

^6^ Ibuprofen selected as example of NSAIDs
