# Supplementary material for: Practical needs and considerations for refugees and other forcibly displaced persons with neurological disorders: Recommendations using a modified Delphi approach
Source: Gates Open Res. 2022 Mar 25;5:178. Originally published 2021 Dec 14. [Version 2] doi: 10.12688/gatesopenres.13447.2 (PMC8901583; doi:10.12688/gatesopenres.13447.2)
Supplement: Peer review history

# Peer review: Reviewer response for version 2

**DOI:** 10.21956/gatesopenres.14864.r31896

**Published:** 2022-03-25

**Authors:** Ibrahem Hanafi

**Affiliations:** 1 Division of Neurology, Department of Internal Medicine, Faculty of Medicine, Damascus University, Damascus, Syria; 2 Department of Neurology, University Hospital of Würzburg and Julius Maximilian University of Würzburg, Würzburg, Germany

Many thanks for addressing my points, and congratulations on your excellent report. I have no further comments.

Is the work clearly and accurately presented and does it cite the current literature?

Yes

If applicable, is the statistical analysis and its interpretation appropriate?

Yes

Are all the source data underlying the results available to ensure full reproducibility?

Yes

Is the study design appropriate and is the work technically sound?

Yes

Are the conclusions drawn adequately supported by the results?

Yes

Are sufficient details of methods and analysis provided to allow replication by others?

Partly

Reviewer Expertise:

Internal medicine, Neurology, Neuroscience, Evidence-based medicine, Deep brain stimulation, Movement disorders

I confirm that I have read this submission and believe that I have an appropriate level of expertise to confirm that it is of an acceptable scientific standard.

# Peer review: Reviewer response for version 1

**DOI:** 10.21956/gatesopenres.14704.r31815

**Published:** 2022-03-07

**Authors:** Ibrahem Hanafi

**Affiliations:** 1 Division of Neurology, Department of Internal Medicine, Faculty of Medicine, Damascus University, Damascus, Syria; 2 Department of Neurology, University Hospital of Würzburg and Julius Maximilian University of Würzburg, Würzburg, Germany

Thank you for considering me for reviewing your high-quality, well-presented manuscript. This novel report is of a great value and conveys important messages to neurologists and organizations working with displaced populations and worldwide. It is well written and provides sufficient details about the methodologies, which robustly support the illustrated conclusions. The panel is limited to nine participants, but they reflect a variety of experiences with displaced people worldwide, which intensify the generalizability of the findings.

 I just have a few suggestions and concerns that I think might answer some doubts from readers when adequately managed.

 Two points were in my opinion overlooked in the introduction, which are the lack of specialized neurologists in areas of conflict and the lack of epidemiological reports published during times of war. For instance, physicians including neurologists in Syrian centers dropped during the first few years of war (Fouad *et al.*, 2017 ^1^), which put more pressure and drained the capacities of the other remaining doctors impacting the quality of care (Mohamad *et al.*, 2021 ^2^ and Hanafi *et al.*, 2021 ^3^).

 Although, esteemed recommendations on increasing neurology experts on the frontlines and telemedicine were raised. Readers might be additionally curious about the opinions of the panel regarding the extent of experience lacking in treating displaced populations, and how they would prioritize increasing the number of neurologists in comparison with the other logistic needs presented on the manuscript. Additionally, one further possible future perspective of the manuscript can be suggesting a modulation of the neurology curriculum of med-schools (e.g., increasing the training hours of more prevalent disorders) in areas where a high percentage of displaced populations is perceptible (Latifeh *et al.*, 2021 ^4^).

 I believe readers with rather limited clinical experience, like me, might wonder about the reason behind the detailed depiction of antiepileptic drugs while several of them can be used interchangeably in places with limited resources. In contrast, I see that the authors combined all basic blood tests in one category. May the authors please provide more information on the reason behind that? Based on that, it is also not very clear to me whether the estimation of population in need (table 4) for the three antiepileptic drugs overlap or sum up?

 I have a minor wonder regarding the following sentence: “An illustrative dosage for each medication was selected based on prescribing patterns that are common for treating neurological conditions in adults.”. Although the illustrative dosage concept is understandable and necessary in such kinds of analysis, I wonder specifically about the long-term medications like antiepileptic drugs. What is generally the time window (i.e., length of therapy) considered when estimating the cost?

 I have another concern regarding the cost estimation of the imaging interventions. The study says they were projected from estimates in European countries. However, more details on that projection can be of high importance to the readers. Would the esteemed opinions of the authors agree that imaging studies required in areas of conflicts should be substantively higher than the ones ordered in the relatively safe Europe during the last few decades? Would they also agree that the ratio CT:MRI scans might deviate for the displaced populations from other stable wealthy populations?

 I would add one point to the limitations that were adequately discussed by the authors. Although the experience of the panel allows them to speak on refugees, asylum seekers, and internally displaced populations (table 3), the article does not present any divergence of the opinions between the three groups, mixing all the views in one category that might be heterogenous regarding some points of the analysis.

 Nevertheless, these suggestions do never affect my recommendations to index this valuable and vital article that drops attention to the neurological care of vulnerable populations and tries to alleviate their suffering worldwide.

Is the work clearly and accurately presented and does it cite the current literature?

Yes

If applicable, is the statistical analysis and its interpretation appropriate?

Yes

Are all the source data underlying the results available to ensure full reproducibility?

Yes

Is the study design appropriate and is the work technically sound?

Yes

Are the conclusions drawn adequately supported by the results?

Yes

Are sufficient details of methods and analysis provided to allow replication by others?

Partly

Reviewer Expertise:

Internal medicine, Neurology, Neuroscience, Evidence-based medicine, Deep brain stimulation, Movement disorders

I confirm that I have read this submission and believe that I have an appropriate level of expertise to confirm that it is of an acceptable scientific standard.

## References

[1] : Health workers and the weaponisation of health care in Syria: a preliminary inquiry for The Lancet –American University of Beirut Commission on Syria. *The Lancet* .2017;390(10111) : 10.1016/S0140-6736(17)30741-9 2516-2526 10.1016/S0140-6736(17)30741-9 28314568

[2] : Workplace violence toward resident doctors in public hospitals of Syria: prevalence, psychological impact, and prevention strategies: a cross-sectional study. *Human Resources for Health* .2021;19(1) : 10.1186/s12960-020-00548-x 10.1186/s12960-020-00548-x 33413470PMC7791675

[3] : Medical research conduct and publication during higher education in Syria: attitudes, barriers, practices, and possible solutions. *Research Square* .2021; 10.21203/rs.3.rs-382753/v2 10.21203/rs.3.rs-382753/v2 PMC945834936092380

[4] : Crisis-based psychiatry curriculum update: A cross-sectional study and an expert reflection from Syria. *Asian J Psychiatr* .2021;61: 10.1016/j.ajp.2021.102681 102681 10.1016/j.ajp.2021.102681 34004462

# Response

**Published:** 2022-03-12

**Authors:** Farrah Mateen

We thank the reviewer for these suggestions. Each of the queries has been addressed in the new version of the manuscript. The section on the update for readers details these changes. Specifically, we updated the introduction to include the very important points that: (1) the epidemiology of neurological disorders are not well documented in humanitarian crises; (2) the number of neurologists in most armed conflicts is very few and may become fewer in times of conflict; (3) conflicts/humanitarian emergencies can be protracted and data may be a snapshot of a more longitudinal process; and (4) health care facilities and workers can be targets of armed conflict. 

 Several references (as recommended) were added to include important data from the Syrian situation.

 The length of time for estimating medicines' costs for chronic conditions (e.g. epilepsy) was one year. 

 The importance of antiseizure medicines was apparent in this consensus process. We include some potential explanations in the paper: (1) the importance of epilepsy as a treatable and disabling condition, and (2) the use of some of these medicines for dual purposes, such as treatment of comorbid pain.

 We have our raw data available on figshare which may provide some elements of heterogeneity in the process. Finally, we clarify that our interventions for prioritization are "concrete" and we did not include purchase of human resources for health (e.g. training a new neurologist) in that prioritization, considering separate from the current process. Thank you for your helpful comments and sharing your knowledge on this topic.

# Peer review: Reviewer response for version 1

**DOI:** 10.21956/gatesopenres.14704.r31816

**Published:** 2022-03-03

**Authors:** Erich Schmutzhard

**Affiliations:** 1 Neurological Intensive Care Unit, Department of Neurology, Medical University Innsbruck, Innsbruck, Austria

In a time, when more than one percent (1%) of the world’s population has to be perceived as forcibly displaced it is highly in time that the medical community, including neurologists from all over the world, are aware and must be made aware of diseases and their emergency diagnostic and therapeutic management steps in order to allow – according to Hippocrates – the best possible management of all those being in need. The authors, working in African, Asian, North- and Central American and European countries clearly show in this manuscript that they are aware of these needs and, even more, are well aware that this knowledge needs to be spread amongst the entire community of neurologists and medical doctors respectively.

 Therefore, I suggest indexing this well-written and highly needed manuscript as it stands.

Is the work clearly and accurately presented and does it cite the current literature?

Yes

If applicable, is the statistical analysis and its interpretation appropriate?

Yes

Are all the source data underlying the results available to ensure full reproducibility?

Yes

Is the study design appropriate and is the work technically sound?

Yes

Are the conclusions drawn adequately supported by the results?

Yes

Are sufficient details of methods and analysis provided to allow replication by others?

Yes

Reviewer Expertise:

Neurology, Tropical Medicine, in particular Tropical Neurology, Emergency and Critical Care Neurology

I confirm that I have read this submission and believe that I have an appropriate level of expertise to confirm that it is of an acceptable scientific standard.

# Response

**Published:** 2022-03-12

**Authors:** Farrah Mateen

The authors thank the reviewer for his review and thoughtful comments.
